# Effect of nutrition education on dietary diversity among HIV Patients in Southeast, Nigeria

**DOI:** 10.4314/ahs.v23i1.19

**Published:** 2023-03

**Authors:** Ifeyinwa Lilian Ezenwosu, Osita Uchenna Ezenwosu

**Affiliations:** 1 Department of Community Medicine, University of Nigeria Teaching Hospital, Enugu State, Nigeria; 2 Department of Paediatrics, University of Nigeria Teaching Hospital, Enugu State, Nigeria

**Keywords:** Nutrition education, dietary diversity, HIV/AIDS

## Abstract

**Background:**

Integrating nutrition interventions which include nutrition education in HIV/AIDS care program may help people living with HIV/AIDS (PLWHA) make better decisions regarding their nutrition to improve their immune system.

**Objective:**

To determine the effect of nutrition education on dietary diversity among HIV/AIDS patients in Southeast, Nigeria.

**Methods:**

A quasi-experimental study was conducted among 370 HIV patients divided into two groups of 185 each for the study and control groups. The nutrition education program was delivered to the study group. In both groups, their practice of dietary diversity was ascertained pre- and post-intervention. The Chi-square test and McNemar were used in the analysis.

**Results:**

Practice of dietary diversity was low among 79(42.7) and 69(37.3) respondents in the study and control groups respectively (x^2^ =1.126, p=0.289). Three months after the intervention, the proportion of respondents with low dietary diversity significantly decreased from 42.7% to 22.7% in the study group while the control group had no appreciable reduction (x^2^=7.532, p=0.006).

**Conclusion:**

Nutrition education plays a positive role in the dietary diversity of PLWHA. This suggests that nutrition education should be a key component in the care of PLWHA for a better nutritional outcome.

## Introduction

Dietary diversity is referred to as the number of different food groups consumed over a reference period (usually 24 hours). It is widely recognized as a key dimension of diet quality, 1 as consumption of food from a variety of groups has the potential of providing the daily nutrient requirement.^2^ People living with HIV/AIDS (PLWHA) when compared to non-infected healthy people require an increased nutrient intake to maintain their immune system.^3^ A non-HIV-infected adult requires an energy intake of approximately 2070 kcal/day and 57 grams/day of protein while an HIV-infected adult requires about 10 to 15% energy increase per day and 50 to 100 % increase in protein in persons of the same age, sex and physical activity level.^4^,^5^

Most HIV infections occur in resource-poor low and middle-income countries across the globe where nutritional problems are common because of the poor pattern of diet intake which is predominantly based on starchy staples and lacks vegetables, fruits and animal source foods.^6^,^7^ This leads to an increased risk of developing micronutrients deficiency, inability to fight opportunistic infections due to poor immune system and an increase in malnutrition-related deaths among PLWHAs.^8^ Thus the need to educate HIV clients on the importance of consuming a better-diversified diet and help them make rational nutritional choices. When the nutrition education gap is filled through food-based approaches using indigenous foods that are highly nutritious and culturally acceptable, there is improvement in the consumption of diversified diets among HIV-infected persons.^9^ With improved nutrition, people living with HIV are likely to: have a better quality of life and thus be able to work and contribute to family income; have prolonged good health and able to care for themselves and help with the care of their children; have reduced illnesses and recover more quickly from infections thus reducing the cost of health care.^10^ Evidence suggests that nutrition education is an essential component in the management of PLWHA but few studies have been done in developing countries including Nigeria among this group to determine its effect on their dietary intake to support or refute this evidence.^11^ Therefore, this study assessed the effect of nutrition education on dietary diversity among HIV patients in Southeast, Enugu state.

## Methods

This was a quasi-experimental study that was conducted among PLWHAs who are above 18 years and access care in HIV clinics at the two tertiary hospitals in Enugu State. The patients at the University of Nigeria Teaching Hospital (UNTH) served as the study group while the patients at the Enugu State University Teaching Hospital (ESUTH) served as the control. The HIV clinics provide promotive, preventive and treatment services to PLWHAs in the state and neighbouring states in Southeast Nigeria. Those included in the study were HIV-positive adults who gave consent and had been on antiretroviral therapy for at least 6 months. Excluded from the study were pregnant or nursing mothers, those who partook and ate in a festive event a day before the questionnaire was administered and HIV patients with other chronic conditions such as cancers, diabetes mellitus, etc. A sample size of 370 patients consisting of 185 each in the study and control groups formed the respondents in the study. The sample size was calculated using the formula for sample size of studies to compare two proportions of equal sizes^12^ as stated below (*Zα + Zβ*)^2^ *X* [(*P*_1_(1 – *P*_1_) + (*P*_2_(1 – *P*_2_)]/(*P*_1_ – *P*_2_)^2^. Where Zα (standard deviate at α probability) =1.96, Zβ (standard deviate at β probability) =0.84, P_1_ (proportion with low dietary diversity) = 0.623^13^, P_2_= 25% expected difference after intervention in the study, and a 10% non-response rate.

The study was carried out in 3 stages: A pre-intervention phase: during which baseline data on dietary diversity were collected from the study and control groups using a semi-structured interviewer-administered questionnaire. The dietary diversity questionnaire was adapted from Food and Agriculture (FAO), 1,14, and was used to assess their 24-hour dietary recall using nine food groups. The client scored 1 for each food group consumed and 0 for each food group not consumed. Dietary diversity score (DDS) was assigned to each patient based on the sum of 9 points and was categorized as low DDS for consumption of 0-4 food groups while ≥5 food groups represented high DDS.

### Intervention Phase

Nutrition education was administered to only the respondents in the study group. The tool for nutrition education was adapted from FAO's Training Manual on Nutrition care for PLWHA^15^,^16^ by considering the different types of foods locally available in the study area. The education was delivered by a dietician as a presentation with supportive visual aids and practical demonstration of the variety of locally available foods one can consume on daily basis. The content of the nutrition education consisted of 4 sessions: session 1 which was on the introduction of dietary diversity, session 2 consisted of basic nutrition with a practical demonstration of the variety of foods using locally available food, session 3 explained the relationship between nutrition and HIV, and session 4 demonstrated the nutritional guide for PLWHA. The education was delivered every day to a cohort of 20 respondents who were recruited on each day for the study and this continued each day of recruitment till the total number of the respondents in the study group were given nutrition education. Thus, the nutrition education was delivered for two months. To enhance participation during the sessions, clients were intermittently asked to summarize what they understood from each session before commencing the next session. At the end of the education on each day, illustrative posters were given to the participants to constantly remind them of what was taught in the programme.

### Post-intervention phase

which involved collection of data after three months on dietary diversity from both the study and control groups using a semi-structured interviewer-administered questionnaire.

### Ethical Considerations

The study was conducted in compliance with the ethical guidelines of the Health Research Ethics Committee of the University of Nigeria Teaching Hospital, Enugu after obtaining ethical clearance (Reference number: UNTH/CSA/329/VOL.5). Permission to conduct the study was obtained from the management of both HIV treatment centres. The respondents gave written informed consent after they had been duly informed of the purpose of the study, the procedure, the pros and cons, their right to the confidentiality of information, possible benefit, risk, and their right to opt-out of the interview or study at any stage.

### Data analysis

Statistical analysis was done using Statistical Package for Social Sciences (SPSS) version 22 (SPSS Inc., Chicago, Illinois). Means and standard deviations of numeric variables were calculated, while frequencies and proportions were generated for categorical variables. The Chi-square test was used to compare proportions as well as to measure associations between categorical variables in both the study and control group. The level of dietary diversity was determined at baseline and 3 months later for study and control groups which were compared using the chi-square test. The effectiveness of nutrition education intervention was determined by comparing the proportion of respondents with high dietary diversity before and after intervention between the study and control groups using a chi-square test while McNemar's chi-square was used to compare proportions within the two groups. The level of statistical significance was set at a p-value <0.05 for all cross-tabulations and inferential analysis.

## Results

### Socio-demographic characteristics of the respondents

The socio-demographic characteristics of the respondents are shown in table 1. A higher proportion of the respondents in both groups were within the age category of 30 and 49 years (65.4% of the study group and 64.4% of the control group) with a mean age ± standard deviation of 42.54 ± 10.447 years and 39.72±10.316 years respectively. The majority of the patients in both groups were female while about half of them were married. Considering the level of education in both groups, 42.7% of the study group and 46.5% of the control group had Secondary education as their highest level of education. The highest proportion of the respondents in the study and control groups (47.0% vs 48.1% respectively) were in business as their occupation.

**Table 1:** Socio-demographic characteristic of the HIV patients in the study and control groups

Variables	Study group	Control group	Test
	n = 185	n = 185	Statistic
	N (%)	N (%)	χ^2^(p-value)
**Age in categories(years)**			
20 – 29	20(10.8)	33(17.8)	5.593(0.133)
30 – 39	57(30.8)	63(34.1)	
40 – 49	64(34.6)	56(30.3)	
≥ 50	44(23.8)	33(17.8)	
**Total**	**185(100)**	**185(100)**	
**Sex**			
Male	51(27.6)	46(24.9)	0.349(0.555)
Female	134(72.4)	139(75.1)	
**Total**	**185(100)**	**185(100)**	
**Marital Status**			
Single	35(18.9)	54(29.2)	5.994(0.050)
Married	95(51.4)	89(48.1)	
Widowed	55(29.7)	42(22.7)	
**Total**	**185(100)**	**185(100)**	
**Highest Educational level**			
No formal education	7(3.8)	2(1.1)	
Primary	49(26.5)	40(21.6)	2.755(0.252)
Secondary	79(42.7)	86(46.5)	
Tertiary	50(27.0)	57(30.8)	
**Total**	**185(100)**	**185(100)**	
**Occupation**			
Civil servants	43(23.2)	52(28.1)	3.936(0.269)
Business	87(47.0)	89(48.1)	
Artisan	35(18.9)	22(11.9)	
Unemployed	20(10.8)	22(11.9)	
**Total**	**185(100)**	**185(100)**	
**Living children**			
0	44(23.8)	64(34.6)	5.248(0.073)
1 – 2	49(26.5)	43(23.2)	
>2	92(49.7)	78(42.2)	
**Total**	**185(100)**	**185(100)**	
**Household size**			
1 – 3	61(33.0)	63(34.1)	4.248(0.120)
4 – 6	82(44.3)	95(51.4)	
>6	42(22.7)	27(14.6)	
**Total**	**185(100)**	**185(100)**	
**Family monthly income (₦)**			
≤ 30,000	85(45.9)	86(46.5)	12.265(0.002)
31,000 – 60,000	43(23.2)	67(36.2)	
> 60,000	57(30.8)	32(17.3)	
**Total**	**185(100)**	**185(100)**	

### The different food groups consumed by the respondents

Table 2 shows that before the intervention, there was no significant difference between the study and control group in the different food groups consumed 24 hours before the interview except in organ meat where a slightly greater proportion of the respondents compared to the control group consumed organ meat. At post-intervention, vitamin A rich fruits and vegetables (p=0.002), other fruits and vegetables (p=<0.001), organ meat (p=<0.001) and legumes (p=0.009) were significantly consumed at a greater proportion in the study group compared to the control group.

**Table 2:** Different food groups consumed by HIV patients

Food groups	Pre-intervention		Test statistics	Post-intervention		Test statistics
	Study group n=185	Control group n=185	χ^2^ (P-value)	Study group n=185	Control group n=185	χ^2^ (P-value)
**Starchy staples**	179(96.8)	181(97.8)	0.411(0.521)	181(97.8)	182(98.4)	0.146(1.000)**
**Dark green vegetables**	141(76.2)	145(78.4)	0.246(0.620)	161(87.0)	153(82.7)	1.347(0.246)
**Vitamin A rich fruits and** **vegetables**	103(55.7)	112(60.5)	0.899(0.343)	136(73.5)	108(58.4)	9.435(0.002)
**Other fruits and** **vegetables**	82(44.3)	90(48.6)	0.695(0.404)	115(62.2)	78(42.2)	14.828(<0.001)
**Organ meat**	9(4.9)	2(1.1)	4.591(0.032)	22(11.9)	2(1.1)	17.823(<0.001)
**Meat/fish**	155(83.8)	156(84.3)	0.020(0.887)	163(88.1)	162(87.6)	0.025(0.874)
**Eggs**	15(8.1)	17(9.2)	0.137(0.711)	21(11.4)	19(10.3)	0.112(0.738)
**Legumes/nuts and Seeds**	143(77.3)	148(80.0)	0.402(0.526)	159(85.9)	139(75.1)	5.473(0.009)
**Milk and milk products**	64((34.6)	57(30.8)	0.602(0.438)	79(42.7)	63(34.1)	2.926(0.087)

** Fisher's test

### Pre and the post-intervention overall practice of dietary diversity

In table 3, the pre-intervention overall dietary diversity practice showed that the mean dietary diversity score was 4.8 with a standard deviation of 1.3 in the study group while the control group had a mean dietary diversity score of 4.9 with a standard deviation of 1.4. There was no statistically significant difference in the overall practice of dietary diversity between the study and control group (χ2= 1.126, p=0.289). Post-intervention findings showed that 77.3% of the respondents in the study group and 64.3% of the control group had high dietary diversity which was statistically significant between the groups (χ2 = 7.532, p =0.006). Also, table 4 shows a statistically significant change within the study group (Mc, p≤0.001) but no difference was noted within the control group regarding their practice of dietary diversity.

**Table 3:** The Pre and post-intervention overall practice of dietary diversity by HIV patients

Dietary diversity Score	Pre-intervention		Test statistics	Post-intervention		Test statistics
	Study group n=185	Control group n=185	χ^2^ (P-value)	Study group n=185	Control group n=185	χ^2^ (P-value)
**Low (0 – 4)**	79(42.7)	69(37.3)	1.126(0.289)	42(22.7)	66(35.7)	7.532(0.006)
**High (5 – 9)**	106(57.3)	116(62.7)		143(77.3)	119(64.3)	
**Total**	**185(100)**	**185(100)**		**185(100)**	**185(100)**	

**Table 4:** Summary of the proportion of respondents with change in the level of practice of dietary diversity within the group at Baseline and after three months

Variable	Study group		Control group	
Dietary diversity	Pre-intervention (n=185)	Post-intervention (n = 185)	Beginning of study (n =185)	End of study (n =185)
<5 (Low)	79(42.7)	42(22.7)	69(37.3)	66(35.7)
≥5 (High)	106(57.3)	143(77.3)	116(62.7)	119(64.3)
**Total**	**185(100)**	**185(100)**	**185(100)**	**185(100)**
	**Mc, p<0.001**		**Mc, p = 0.818**	

## Discussion

Dietary diversity is considered a crucial component of comprehensive care for PLWHA particularly in poor resource settings where food insecurity is an endemic problem and low-quality monotonous diets are the norm.^17^ Despite the importance of dietary diversity in HIV patients, its practice may be affected by lack of exposure to nutrition education and this may lead to poor consumption of high-quality diet which subsequently affects their ability to fight opportunistic infections due to poor immune system.^18^

Regarding the practice of dietary diversity at baseline, slightly above half of the respondents in both groups had a high dietary diversity score which was similar to the findings in Uganda^19^ and Kenya.^20^ In contrast, Akwiwu and Akinbile^13^ in Imo State Nigeria found that the majority of their respondents had low dietary diversity. This difference in finding may be due to different study settings as the study in Imo State^13^ was community-based while this study was facility-based. Patients in health facilities tend to have access to an informal form of nutrition education during counselling which may impact dietary practice. Despite the differences in their dietary diversity scores, this study and earlier studies^13^,^19^,^21^ noted that the majority of foods consumed by the HIV patients over 24 hours were starchy staples. This emphasizes the long-held fact that most resource-poor, low and middle-income countries (LMIC) consume foods predominantly based on starchy staples^17^,^21^ and underscores the need for nutritional restructuring away from starchy-based foods in these LMICs. On the other hand, the food group least consumed in this study was organ meat. A similar finding was noted by earlier studies.^21^,^22^ Compared to other parts of animal meat, organ meat may not be numerous enough to be readily available and with possible high cost, people in low-income areas may be facing affordability challenges

Three months after the intervention, the result of this study revealed a significant increase in the proportion of respondents with high dietary diversity practice among the study group in comparison with the control group which had no improvement in their dietary diversity practice. In addition, there was a significant increase in the proportion of clients who ate foods rich in the following: dark green vegetables, vitamin A rich fruits, other fruits and vegetables, organ meat and legumes/nuts and seeds over 24 hours among the study group compared to control group. These findings were comparable with previous interventional studies that reported an increase in the level of practice post-intervention.^23^,^24^,^25^ This implies that nutrition education is an important strategy that should be continuously delivered throughout the point of care of PLWHA.

## Conclusion

Following nutrition education, a significant proportion of the study group showed a positive increase in the practice of dietary diversity when compared to the control group who were not exposed to the contents of the training. Therefore, Nutrition education should be a key component in the care of PLWHA and should be initiated at the entry point to comprehensive care. It should also be continuous throughout care. It is, therefore, suggested that information, education and communication materials such as posters, fliers, songs, etc on food groups with their sources and functions should be conspicuously displayed and sung in the HIV clinics to aid the practice of dietary diversity.
